# Evaluation of Traumatic Spinal Cord Injury in a Rat Model Using ^99m^Tc-GA-5 as a Potential In Vivo Tracer

**DOI:** 10.3390/molecules26237138

**Published:** 2021-11-25

**Authors:** Vanessa Izquierdo-Sánchez, Pablo C. Zambrano-Rodríguez, Nadia Peña-Merino, Sirio Bolaños-Puchet, Horacio J. Reyes-Alva, Angelina Martínez-Cruz, Saé Muñiz-Hernández, Gabriel Guízar-Sahagún, Luis Alberto Medina

**Affiliations:** 1 Unidad de Investigación Biomédica en Cáncer INCan/UNAM, Instituto Nacional de Cancerología, Ciudad de México 14080, Mexico; van.izq.san@gmail.com (V.I.-S.); nadiamerp@gmail.com (N.P.-M.); seirios@ciencias.unam.mx (S.B.-P.); 2 Department of Neurology, Facultad de Medicina Veterinaria y Zootecnia, Universidad Autónoma del Estado de México, Toluca 50090, Mexico; drpablozambrano@gmail.com (P.C.Z.-R.); reyeshavet@gmail.com (H.J.R.-A.); 3 Facultad de Ciencias Veterinarias, Universidad Técnica de Manabí, Portoviejo 130105, Manabí, Ecuador; 4 Facultad de Estudios Zaragoza, Universidad Nacional Autónoma de México (UNAM), Ciudad de México 09230, Mexico; 5 Department of Experimental Surgery, Proyecto Camina A.C., Calzada de Tlalpan 4430, Ciudad de México 14050, Mexico; markim_00@yahoo.com.mx; 6 Laboratorio de Oncología Experimental, Subdirección de Investigación Básica, Instituto Nacional de Cancerología, Ciudad de México 14080, Mexico; sayide@hotmail.com; 7 Research Unit for Neurological Diseases, Hospital de Especialidades Centro Médico Nacional Siglo XXI, IMSS, Ciudad de México 06720, Mexico; 8 Instituto de Física, Universidad Nacional Autónoma de México (UNAM), Ciudad de México 04510, Mexico

**Keywords:** spinal cord injury, GFAP, GA-5 monoclonal antibody, ^99m^Tc-GA-5, microSPECT/CT imaging, astrogliosis, molecular imaging

## Abstract

Spinal cord injury (SCI) refers to the damage suffered in the spinal cord by any trauma or pathology. The purpose of this work was to determine whether ^99m^Tc-GA-5, a radiotracer targeting Glial Fibrillary Acidic Protein (GFAP), can reveal in vivo the reactivation of astrocytes in a murine model with SCI. A method for the ^99m^Tc radiolabeling of the mouse anti-GFAP monoclonal antibody GA-5 was implemented. Radiochemical characterization was performed, and radioimmunohistochemistry assays were used to evaluate the integrity of ^99m^Tc-GA-5. MicroSPECT/CT was used for in vivo imaging to trace SCI in the rats. No alterations in the GA-5’s recognition/specificity ability were observed after the radiolabeling. The GA-5’s radiolabeling procedure implemented in this work offers a practical method to allow the in vivo following of this monoclonal antibody to evaluate its biodistribution and specificity for GFAP receptors using SPECT/CT molecular imaging.

## 1. Introduction

Traumatic spinal cord injury (SCI) is a public health problem that affects the economically active population, leading to neurological, sensory, and motor deficits [1,2,3,4]. The World Health Organization (WHO) estimates worldwide incidence varies between 40 and 80 cases per million habitants [1]. Long-term neurological recovery continues to be limited, producing physical, emotional, and economic consequences for patients, families, and society. To date, effective therapies for SCI remain challenging, which has encouraged an exhaustive search focused on the characterization of the pathophysiology of this condition and the development of innovative therapeutic methods that aim to repair the injured spinal cord [5,6].

The Glial Fibrillary Acidic Protein (GFAP) is the main structural protein of the filaments within the cytoskeleton of astrocytes and acts as a marker of mature astrocytes. The roles attributed to GFAP in the Central Nervous System (CNS) include the suppression of neuronal proliferation and neurite extension in the mature brain, formation of a physical barrier to isolate damaged tissue, participation in cerebellar motor learning, blood flow regulation following ischemia, participation in the blood-brain barrier, supporting myelination, and providing mechanical strength [7]. Recent evidence suggests that GFAP and its breakdown products are rapidly released into biofluids after traumatic brain and spinal cord injuries and stroke, making them robust candidate biomarkers for such disorders [8]. Consequently, GFAP biomarkers can be further developed into theranostic molecules that can help to treat and diagnose CNS injury.

Astrocytes are the most abundant cell type in the mammalian CNS [9]. After SCI, astrocytes become reactive at the site of injury and beyond, a process known as astrogliosis, characterized by profound morphological, molecular, and functional changes in astrocytes. Astrogliosis can be demonstrated by immunohistochemistry using primary antibodies to GFAP [9,10,11]. Reactive astrocytes are an essential part of the multiple cellular and extracellular components of the glial scar that forms due to the cascade of inflammatory and pathological processes triggered by the initial mechanical injury to the spinal cord [12,13,14].

Molecular imaging technologies such as SPECT, PET, MRI, and optical imaging (i.e., bioluminescence imaging), make it possible to detect in vivo cellular and molecular aspects of specific targets in a spatial-temporal way [15]. In contrast to conventional diagnostic images, molecular imaging represents an emerging field that integrates molecular biology, chemistry, and radiology, giving an effective way to monitor therapeutic strategies, as well as to study physiological and pathological processes at the molecular level of multiple fields, including of course SCI research [16,17,18,19].

Considering the lack of molecular imaging studies to characterize the reactive astrocytic response in the rodent spinal cord after SCI, the present study was designed to test the hypothesis that the use of a radiolabeled antibody targeting GFAP after SCI will allow obtaining molecular images that translate the occurrence of astrogliosis due to the injury. Here, we report the procedures for the technetium-99m (^99m^Tc) radiolabeling of the mouse anti-GFAP monoclonal antibody ((GA-5): sc-58766). The GA-5 reacts with the GFAP, and it is commonly used in immunohistochemical studies to identify astrocytes and other glial cells. Here, SPECT/CT molecular imaging procedures were implemented to evaluate the ^99m^Tc-GA-5 (^99m^Tc-anti-GFAP) as an in vivo radiotracer in a rat SCI model.

## 2. Results

### 2.1. Anti-GFAP Radiolabeling and Integrity

**Figure 1:**
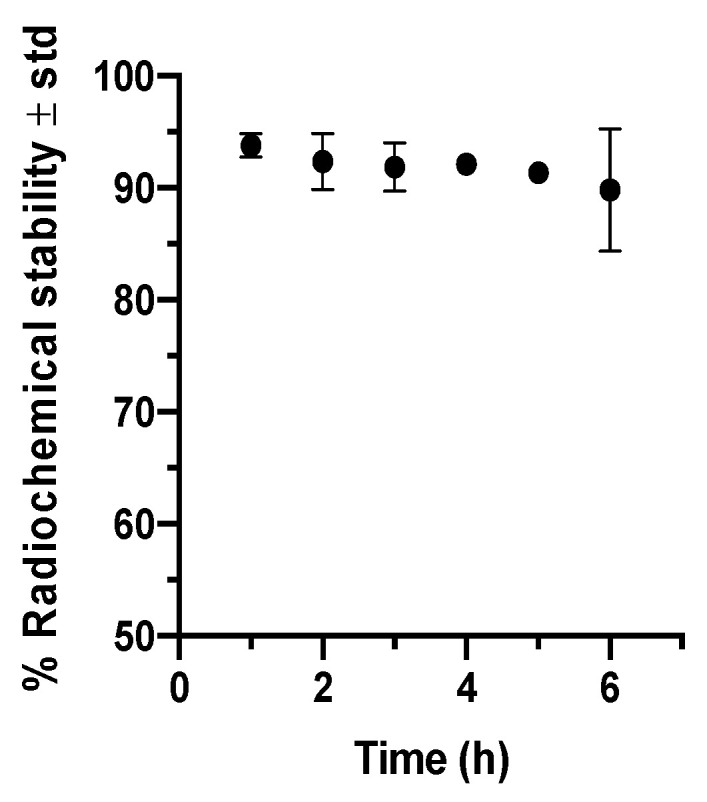
Radiochemical stability of ^99m^Tc-GA-5 in serum during 6 h. Each time-point represents the average values of three independent repetitions. No statistical differences were observed between each point.

Radiolabeling efficiency (RE) and Radiochemical purity (RCP) percentages were 68.9 ± 9.4 and 93.4 ± 3.4, respectively (n = 5); RE was enough to perform the imaging studies, and the RCP indicates an efficient radiolabeling process. Figure 1 illustrates radiochemical stability around 90% in serum during 6 h incubation, suggesting ^99m^Tc-GA-5 for in vivo imaging studies to trace SCI. 

**Figure 2:**
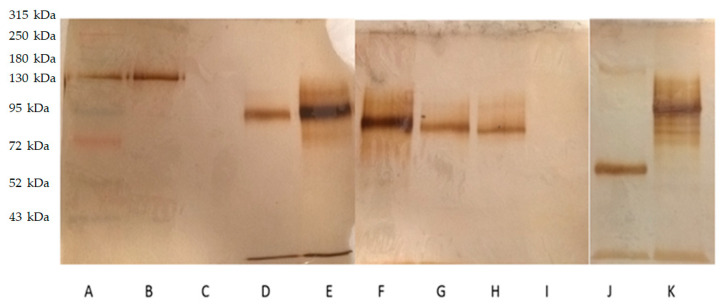
SDS-PAGE. (**A**) Molecular weight size marker; (**B**) GA-5; (**C**) residuals after GA-5 filtering in Amicon columns; (**D**) GA-5 after reduction in disulfide bridges; (**E**) GA-5/MDP; (**F**) ^99m^Tc-GA-5 (RIC); (**G**) ^99m^Tc-GA-5 after first purification; (**H**) ^99m^Tc-GA-5 after second purification; (**I**) Draximage-MDP (MDP); (**J**,**K**) GA-5 in reducing and semi-reducing conditions.

^99m^Tc-GA-5 integrity was evaluated with SDS-PAGE (Figure 2); no antibody fragments were found, showing that the methylene diphosphonate (MDP) or the labeling with ^99m^Tc affects Ga-5 integrity. However, GA-5, with a molecular weight of 50 kDa, results in a band of 130 kDa in the SDS-PAGE. This result suggests glycosylation of GA-5, leading to a higher molecular weight. The same situation was observed with Ga-5 in reduction conditions. Columns D–H show higher molecular weight during each step of the radiolabeling process (reducing disulfide bridges, adding the exchanger ligand, and the radionuclide, respectively). Columns J–K show GA-5 in reducing and semi-reducing conditions.

### 2.2. Radioimmunohistochemistry Results

**Figure 3:**
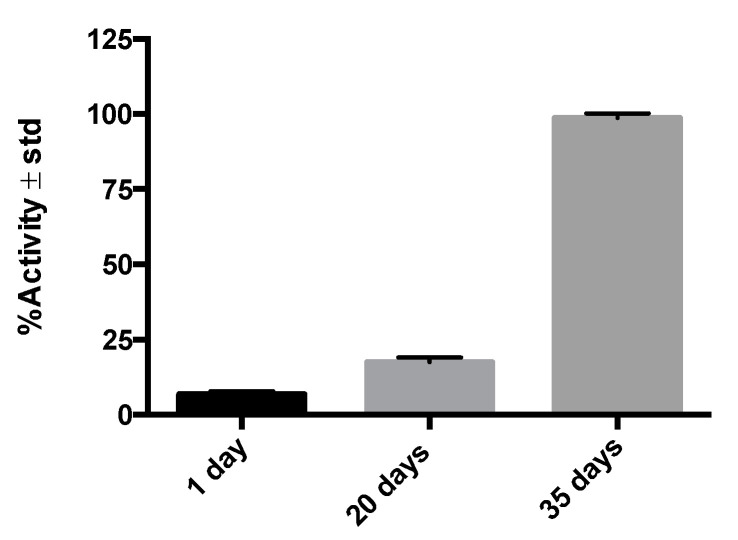
%Activity in post-injury histological samples (n = 3–6) on different days, illustrating the affinity of ^99m^Tc-GA-5 for its receptors in cells of murine medullary tissue.

Figure 3 shows the activity (in counts per minute) in post-injury histological samples on different days, illustrating the ^99m^Tc-GA-5 (RIC) affinity for its receptors in cells of murine medullary tissue as a function of time. The result shows that the expression of GFAP varies to the stage of the SCI, showing that the shorter the post-injury time, the lower the expression of GFAP. The overexpression of GFAP is proportional to the number of reactive astrocytes after the injury. This result shows that the radiolabeling does not affect the GA-5 affinity for its receptors. The controls (tissues of rats without SCI) allow for establishing that the radiolabeled GA-5 does not bind to healthy medullary tissue. 

### 2.3. In Vivo Biodistribution of ^99m^Tc-GA-5 in a Model of SCI

**Figure 4:**
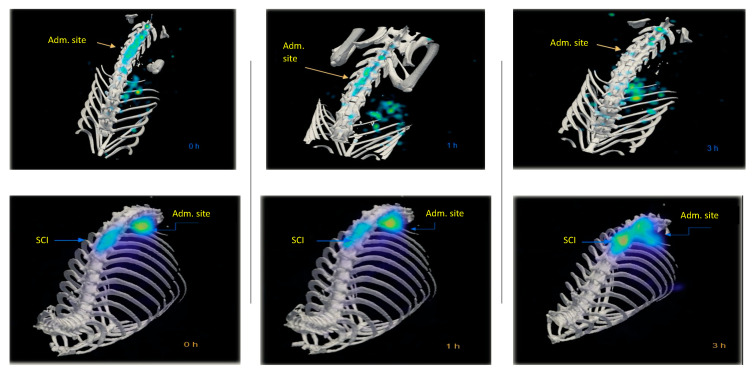
Representative images of rats without (**top**) and with (**bottom**) SCI depicting the biodistribution and accumulation of the ^99m^Tc-GA-5 from the administration site at different times (0, 1, and 3 h).

**Figure 5:**
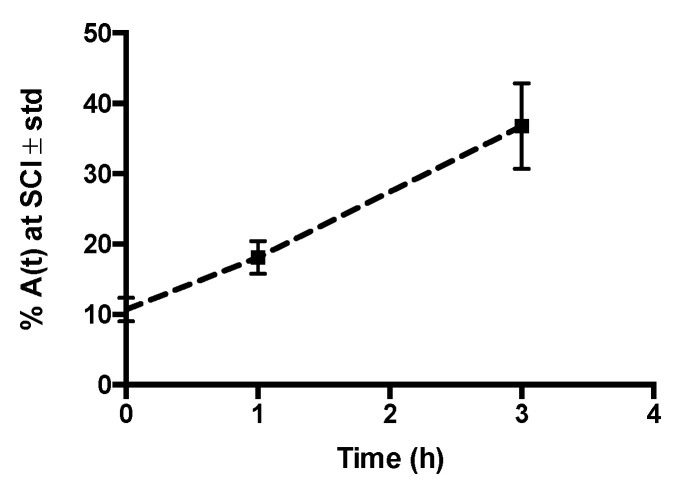
Percentage of ^99m^Tc-GA-5 at the SCI of the rats (n = 3). A continuous accumulation was observed during the three hours of the study.

**Figure 6:**
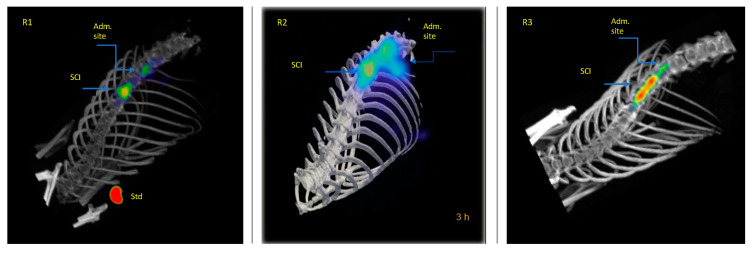
Specific accumulation of ^99m^Tc-GA-5 at the SCI after 3 h post-administration in each rat of the study group.

Figure 4 compares the biodistribution/accumulation of ^99m^Tc-GA-5 at different times in representative rats with and without SCI. In rats without lesions (top images), a rapid movement from the administration site into the liver was observed. In rats with SCI (bottom images), rapid movement from the administration site to the lesion was observed. It was noticed that ^99m^Tc-GA-5 concentrates at the lesion, depicting the RIC’s specific affinity for reactive astrocytes at and around the site of injury. Figure 5 quantitively illustrates this accumulation at different times after the administration; after 3 h, accumulation of ^99m^Tc-GA-5 at the injury was around 37%. Figure 6 shows images of the RIC’s accumulation in each rat with SCI evaluated with ^99m^Tc-GA-5.

**Figure 7:**
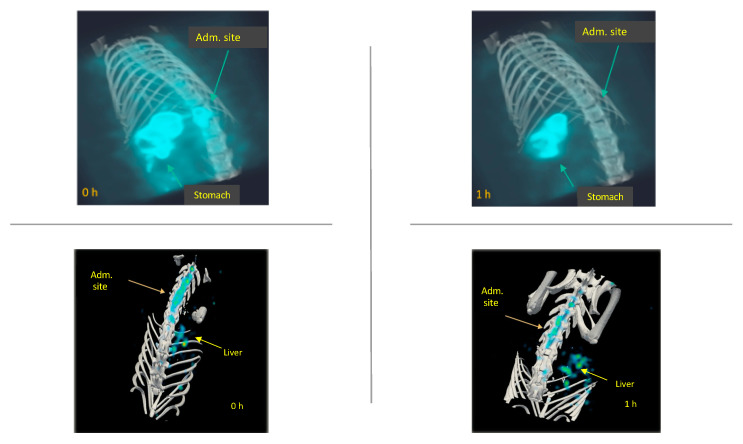
Representative images of rats without SCI depicting differences in the biodistribution of ^99m^Tc (**top**) and ^99m^Tc-GA-5 (**bottom**).

To illustrate the differences in biodistribution between ^99m^Tc and ^99m^Tc-GA-5, Figure 7 depicts representative images of rats without SCI with both radiopharmaceuticals. Fast elimination of the ^99m^Tc from the administration site was observed, compared with ^99m^Tc-GA-5, with accumulation in the stomach. On the contrary, ^99m^Tc-GA-5 quickly accumulates in the liver.

## 3. Discussion

Here, a molecular imaging protocol served as a platform to test the capacity of ^99m^Tc-GA-5 (a radiolabeled anti-GFAP) to identify in vivo the astrocytic response in the spinal cord of rats subjected to SCI with a model that reproduces human SCI [20,21]. 

To accomplish this goal, before performing the imaging studies, we made sure that the radiolabeling efficiency and the radiotracer integrity met the required standards. We also confirmed, by radioimmunohistochemistry, that the RIC is bound to the cells and tissues of interest in a post-injury time-dependent manner. After solving these basic precepts, we designed the imaging protocol grounded on SPECT utilizing a dedicated small animal system (Albira small animal microPET/SPECT/CT imaging system). As a global result, our radiotracer selectively accumulates in glial cells (targeting primarily reactive astrocytes) as an active agent, facilitating the in vivo monitoring of astrogliosis.

GA-5′s receptors can be over or underexpressed in cells and tissues under physiological or pathological conditions and used as molecular targets [15]. The specific interaction between the receptor and its ligand (GFAP and ^99m^Tc-anti-GFAP in our study) proved to be an effective strategy to evaluate this radiopharmaceutical’s amount and residence time in target tissues. By considering the temporal/spatial expression of GFAP in the current radioimmunohistochemistry assay (see Figure 3) and previous histological studies [22], we chose to perform our imaging study at 20 days after injury, as this is the timeframe where reactive astrocytes overexpress GFAP in the context of the glial scar formation. 

Contrary to most in vivo molecular imaging studies where the radioactive biomarker is administered intravenously, we used the intrathecal route successfully. When macromolecules, such as proteins, are administered intravenously, the blood-brain barrier limits their access to the CNS. However, when delivered intrathecally, macromolecules circumvent this barrier and effectively reach their target within the CNS [23,24,25]. The imaging strategy tested here was effective in revealing the cellular and molecular processes sought. The biomarker appears to be highly sensitive to GFAP expression changes and the distribution of the reactive astrocytes at and around the injury site. 

SCI is a pathology with high physical, social, and economic repercussions [1,2,3,26,27,28,29] commonly occurring in vehicular accidents, sports-related injuries, or violent episodes, for which no therapies to restore neurologic deficit exist effectively. After an injury occurs, death of neurons and glial cells, ischemia, and inflammation, which is followed by the formation of a glial scar and cystic cavities in the spinal cord, are observed [30]. Due to the profound impact of astrogliosis on the progression of SCI, a better understanding of the cellular and molecular events in glial scarring is mandatory [31,32]. To date, the role of astrogliosis has been oversimplified in binary terms as good or bad [11,14]. It is considered beneficial because it aids in repairing the initial damage, stabilizes the spread of injury, and fosters axonal regeneration and functional recovery, but detrimental because it provides both a physical and chemical barrier to regenerating axons [7,13,16,31,32]. 

There is no diagnostic strategy for in vivo selective identification of GFAP present in reactive astrocytes through molecular imaging methods. Here, we demonstrate that the rapid identification of astrogliosis through molecular imaging in a murine SCI model is feasible without characterizing the mechanisms that regulate astrocyte reactivity and scar formation. We consider that the main contribution of this study lies in the development of a tool that will be useful to understand better and monitor astrogliosis, a controversial topic of the most significant relevance in the pathophysiology of SCI. 

However, our study has several limitations. Here, we show the sensitivity of the ^99m^Tc-GA-5 in detecting astrogliosis associated with SCI on day 20 after injury; but, to validate this technology in animal models of varying stages after injury (earlier and later), more experiments are necessary since astrogliosis is a dynamic process. Besides, while our data included radioimmunohistochemistry to validate the tracer’s specificity, we did not correlate the SPECT image with the GFAP immunohistochemistry of the corresponding specimens. Thus, future studies are required to elucidate these unresolved issues.

The current article provides a novel complementary identification method and those already known to help optimize treatment for such pathology. Nuclear imaging (SPECT and PET scans) is widely used in cardiology, neurology, and oncology. Although no studies have been reported evaluating molecular imaging technologies targeting GFAP in SCI, some recent studies have reported the utility of nuclear imaging to reveal in vivo biological activity of events associated with SCI, such as acute inflammation in a rodent model [19]. We predict that future improvement in molecular imaging, i.e., PET with long half-life radionuclides, will make essential contributions to our understanding of SCI pathophysiology and assess the therapeutic effect on a molecular scale.

## 4. Materials and Methods

### 4.1. Anti-GFAP Radiolabeling

Radiolabeling was performed using the Schwarz method [33] with some modifications. Briefly, 100 µL of anti-GFAP (GA-5) sc-58766 (50 kDa, 0.2 mg/mL) (Santa Cruz Biotechnology, Inc., Dallas, TX, USA) was reduced by reaction with 1 µL of 2-mercaptoetanol (2-ME) (Sigma-Aldrich Co., St. Louis, MO, USA) at room temperature for 30 min. The excess was removed by centrifugation (5 min, 7000× *g*) using an Amicon Ultra-0.5 filter unit and sterile saline solution. A methylene diphosphonate (MDP) kit (containing 10 mg of medronic acid, 2 mg of para-aminobenzoic acid, and 1.1 mg of stannous chloride dihydrate) (Jeubilant Draximage, Inc., Kirkland, QC, Canada) was dissolved in 5 mL of sterile saline solution purged with nitrogen and 40 µL were mixed with the GA-5 solution at room temperature for 30 min. Then, 185 MBq (5 mCi) of ^99m^Tc pertechnetate was added and gently mixed; the resultant radioimmunoconjugate (RIC) was kept at room temperature for 1 h and purified twice with an Amicon Ultra-0.5 to remove the free ^99m^Tc pertechnetate. The radiolabeling procedure was performed in a Biohazard Laminar Air Flow Hood (Nuaire, Plymouth, MN, USA); glassware, materials, and solutions for the labeling procedure were sterile, pyrogen, and metal-free.

Radiolabeling efficiency (RE) and radiochemical purity (RCP) were estimated by instant thin-layer chromatography on silica-impregnated glass fiber sheets (iTLC-SG) (Agilent Technologies, Santa Clara, CA, USA). Two microliters of RIC were spotted on 10 cm strips of the chromatography sheet paper; methyl ethyl ketone (MEK) and ethanol:ammonium hydroxide:water buffer (2:1:5 [*v*/*v*]) were used as mobile phases to measure %RE and %RCP. The strips were cut in half, and the radioactivity in each segment was measured using a well-type gamma counter (Ludlum 2200, Sweetwater, TX, USA). With MEK, colloidal forms and technetium complex remains at the bottom of the strip and free ^99m^Tc pertechnetate migrates to the top, allowing to estimate the %RE. With the ethanol:ammonium hydroxide:water buffer, free ^99m^Tc pertechnetate and technetium complex migrate with the solvent front, while reduced, hydrolyzed ^99m^Tc remains at the bottom, allowing to quantify the %RCP. The %RE and %RCP were calculated as:

*%RE* = 100 × [*B*_1_/(*T*_1_
*+ B*_1_)]

*%RCP* = 100% − [*T*_1_ + *B*_2_]%

where *B*_1_ and *T*_1_ represent the radioactivity (cps) measured at the bottom and top iTLC-SG segments used with MEK, respectively, and *B*_2_ the radioactivity measured at the bottom segment used with ethanol:ammonium hydroxide:water buffer. Independent iTLC for ^99m^Tc pertechnetate was used as a control. Radiochemical stability was determined after incubation of RIC with fresh human serum (1 mL) at 37 °C for 6 h, and the dissociation of ^99m^Tc was evaluated at different times by the iTLC method described above.

### 4.2. GA-5 Integrity Assay

After radiolabeling, RIC samples were collected and mixed with the loading buffer (glycerol, sodium dodecyl sulfate (SDS) in Tris-HCl 0.5 M pH 6.8) in non-reducing or reducing conditions (2-mercaptoethanol). Electrophoresis was performed on SDS-polyacrylamide (SDS-PAGE) in a Biorad Electrophoretical System (Mini-Protean Tetra Vertical Electrophoresis Cell, Hercules, CA, USA) using 7.5% acrylamide/bis-acrylamide gel. GA-5 in semi-reducing conditions was used as the control. Silver nitrate was used to stain the gel. Additionally, a western blot was performed to determine the GA-5 specificity and evaluate if the recognition regions remained intact.

### 4.3. Animals

Healthy male Long-Evans rats, 10–12 weeks old, were used in this study. The animals were organized into four groups (n = 3/group) of study as Groups A and B (animals with SCI evaluated with either ^99m^Tc-GA-5 or ^99m^Tc) and Groups C and D (animals without SCI evaluated with either ^99m^Tc-GA-5 or ^99m^Tc) and used in the imaging study. Another group (n = 9) of animals with SCI was used for the immunohistochemistry study. Animals were kept in a pathogen-free environment and fed with autoclaved food and water *ad libitum*. A local institutional Ethics Committee approved the procedures for care and use of the animals at Instituto Mexicano del Seguro Social (R-2014-785-099). The Guide for the Care and Use of Laboratory Animals of the NIH (USA) guidelines were also followed. Efforts were made to minimize animal suffering and to minimize the number of animals used.

### 4.4. Traumatic Spinal Cord Injury

Animals were intramuscularly anesthetized with ketamine and xylazine (8 mg/kg). Laminectomy was performed aseptically at the base of the ninth thoracic (T9) spinous processes maintaining meninges intact. The SCI was produced using a New York University impactor (MASCIS impactor) after dropping a 10 g rod from a height of 50 mm, which results in a severe injury. Post-surgical care included manual press of bladders twice a day until bladder function returned. Food and water were provided ad libitum. Ciprofloxacin lactate (8 mg/kg) (Bayer, Mexico City, Mexico) was used as a prophylactic for infections, given subcutaneously every 12 h, starting at the end of surgery, for seven consecutive days. Acetaminophen (Cilag, Mexico) (64 mg/kg/day) was given in the drinking water for one week to prevent self-mutilation. After the injury, the animals were kept for recovery, and then the GFAP expression was evaluated either by in vitro radioimmunohistochemistry or in vivo microSPECT imaging. Sham-injured animals only underwent soft tissue surgery without laminectomy or SCI.

### 4.5. Radioimmunohistochemistry Assay

The animals were anesthetized and euthanized on days 1, 20, and 35 after injury. An intracardiac puncture fixed them. Initially, they were perfused with 200 mL saline, followed by 500 mL 10% buffered formaldehyde. Spine blocks from T8 to T12 were dissected and post-fixed for four days in the same fixative. These blocks were then placed in a 12% ethylenediaminetetraacetic acid (EDTA) aqueous solution (pH 6.5) for decalcification at room temperature. Spinal cuts from rats without injury were used as control. The EDTA solution was changed every day until the vertebrae became soft (around ten days). For three days, decalcified spines were placed in phosphate-buffered saline (PBS) solutions of sucrose ascending concentrations (7.5%, 15%, and 30%) at 4 °C. Spine blocks were cut into individual vertebral segments from T9 to T11; each segment was in turn cut into transverse 5–6 mm thick sections and included in paraffin until the radioimmunohistochemistry assay.

Deparaffinized slides were maintained in humidity chambers (Biolegend^®^), and 10 μL of ^99m^Tc-GA-5 (~5 MBq) were added over each slide and maintained in orbital agitation for 1 hour; then the slides were washed with PBS under agitation for 5 min. The percentage of activity (cpm) in each slide was measured with the well-type counter scintillation.

### 4.6. MicroSPECT Imaging Procedures

Images were acquired using an Albira small animal microPET/SPECT/CT imaging system (Bruker, Spain). Twenty days after SCI or sham-injury, animals were anesthetized by inhalation with isoflurane (2–3% in 100% oxygen) and intrathecally administered with 9.25 MBq (250 μCi) of either ^99m^Tc-GA-5 or ^99m^Tc in a volume of 120 μL of saline solution as previously described [34]. Briefly, anesthetized animals were subjected to a laminectomy in the thoracolumbar region to insert a cannula in the subarachnoid space; then, the radiotracer was administrated within 2 min using an infusion pump. Images were acquired at 0, 1, and 3 h post-administration, and SPECT scans using a pinhole collimator were taken with 30 projections per detector at 60 s/projection. CT scans were performed with 600 projections (tube voltage 45 kV and 0.4 mA). All images were reconstructed with Albira’s reconstruction software based on the ordered subsets expectation-maximization (OSEM) routine (three iterations).

The percentage of activity at some specific time *t* (%A(*t*)) in the lesion site was calculated from the images as the ratio of the detected counts in the lesion and the total number of counts detected in the whole body at baseline (A_0_), multiplied by 100 (%A(*t*)= A_lesion_(*t*)/A_0_ × 100). A volume of interest (VOI) was drawn around the lesion site and the entire body on the images using the PMODE software (PMOD-Tecnologies LLC, Zurich) to determine the number of counts held. Known activity (5 uCi) of ^99m^Tc, contained in an Eppendorf tube located close to the rat during imaging, was used as a reference in the images to correlate the number counts in the VOIs with the activity. Counts associated with the VOIs were corrected for radioactive decay with the image acquisition time.

### 4.7. Statistical Analysis 

Values are reported as mean values ± SEM. Statistical analysis was performed using a one-way analysis of variance (ANOVA) on the SPSS Base 20.0 software (SPSS Inc., Chicago, IL, USA). The significance was determined at *p* < 0.05.

## 5. Conclusions

The GA-5’s radiolabeling procedure implemented in this work offers a practical method to allow the in vivo following of this monoclonal antibody to evaluate its biodistribution and specificity for GFAP receptors using SPECT/CT molecular imaging. No alterations in the GA-5’s recognition/specificity ability were observed after the radiolabeling. An essential finding of this study was the SPECT/CT imaging ability to recognize the overexpression of the GFAP in the SCI model compared with animals without SCI. This finding, observed since the administration, allows a rapid method to evaluate and identify the lesion site and its astrogliosis stage.

## Data Availability

Not applicable.

## Sample Availability

Samples of the compounds are not available from the authors.

## References

[1] OMS Lesiones Medulares. http://www.who.int/mediacentre/factsheets/fs384/es/.

[2] NINDS Spinal Cord Injury Information Page. https://www.ninds.nih.gov/Disorders/All-Disorders/Spinal-Cord-Injury-Information-Page#disorders-r1.

[3] Ruiz A.D., Sahagún G.G., Castañeda C.R. (2002). Estrategias neuroprotectoras después de una lesión traumática de la médula espinal. Rev. Med. IMSS.

[4] NSCISC National Spinal Cord Injury Statistical Center https://www.nscisc.uab.edu/.

[5] Hernández J., Torres-Espín A.N. (2011). Adult stem cell transplants for spinal cord injury repair: Current state in preclinical research. Curr. Stem Cell Res. Ther..

[6] Cizkova D., Murgoci A.N., Cubinkova V., Humenik F., Mojzisova Z., Maloveska M., Cizek M., Fournier I., Salzet M. (2020). Spinal cord injury: Animal models, imaging tools and the treatment strategies. Neurochem. Res..

[7] Brenner M. (2014). Role of GFAP in CNS injuries. Neurosci. Lett..

[8] Yang Z., Wang K.K.W. (2015). Glial fibrillary acidic protein: From intermediate filament assembly and gliosis to neurobiomarker. Trends Neurosci..

[9] Magaki S.D., Williams C.K., Vinters H.V. (2018). Glial function (and dysfunction) in the normal & ischemic brain. Neuropharmacology.

[10] Pekny M., Pekna M. (2004). Astrocyte intermediate filaments in CNS pathologies and regeneration. J. Pathol..

[11] Karimi-Abdolrezaee S., Billakanti R. (2012). Reactive astrogliosis after spinal cord injury-beneficial and detrimental effects. Mol. Neurobiol..

[12] Yuan Y.-M., He C. (2013). The glial scar in spinal cord injury and repair. Neurosci. Bull..

[13] Okada S., Hara M., Kobayakawa K., Matsumoto Y., Nakashima Y. (2018). Astrocyte reactivity and astrogliosis after spinal cord injury. Neurosci. Res..

[14] Bradbury E.J., Burnside E.R. (2019). Moving beyond the glial scar for spinal cord repair. Nat. Commun..

[15] Reubi J.C., Maecke H.R. (2008). Peptide-based probes for cancer imaging. J. Nucl. Med..

[16] Lo W.C., Hsu C.H., Wu A.T., Yang L.Y., Chen W.H., Chiu W.T., Lai W.F., Wu C.H., Gelovani J.G., Deng W.P. (2008). A novel cell-based therapy for contusion spinal cord injury using GDNF-delivering NIH3T3 cells with dual reporter genes monitored by molecular imaging. J. Nucl. Med..

[17] Song F., Tian M., Zhang H. (2014). Molecular imaging in stem cell therapy for spinal cord injury. Biomed. Res. Int..

[18] LeRoux L.G., Bredow S., Grosshans D., Schellingerhout D. (2014). Molecular imaging detects impairment in the retrograde axonal transport mechanism after radiation-induced spinal cord injury. Mol. Imaging Biol..

[19] Albadawi H., Chen J.W., Oklu R., Wu Y., Wojtkiewicz G., Pulli B., Milner J.D., Cambria R.P., Watkins M.T. (2017). Spinal cord inflammation: Molecular Imaging after Thoracic Aortic Ischemia reperfusion injury. Radiology.

[20] Kjell J., Olson L. (2016). Rat models of spinal cord injury: From pathology to potential therapies. Dis. Model. Mech..

[21] Minakov A.N., Chernov A.S., Asutin D.S., Konovalov N.A., Telegin G.B. (2018). Experimental models of spinal cord injury in laboratory rats. Acta Nat..

[22] Baldwin S.A., Broderick R., Blades D.A., Scheff S.W. (1998). Alterations in temporal/spatial distribution of GFAP- and vimentin-positive astrocytes after spinal cord contusion with the New York University Spinal cord injury device. J. Neurotrauma.

[23] Nestrasil I., Shapiro E., Svatkova A., Dickson P., Chen A., Wakumoto A., Ahmed A., Stehel E., McNeil S., Gravance C. (2017). Intrathecal enzyme replacement therapy reverses cognitive decline in mucopolysaccharidosis type I. Am. J. Med. Genet. Part A.

[24] Ineichen B.V., Schnell L., Gullo M., Kaiser J., Schneider M.P., Mosberger A.C., Good N., Linnebank M., Schwab M.E. (2017). Direct, long-term intrathecal application of therapeutics to the rodent CNS. Nat. Protoc..

[25] Wahl A.-S., Correa D., Imobersteg S., Maurer M.A., Kaiser J., Augath M.A., Schwab M.E. (2020). Targeting Therapeutic antibodies to the CNS: A comparative study of intrathecal, intravenous, and subcutaneous Anti-Nogo a antibody treatment after stroke in rats. Neurotherapeutics.

[26] Harkey H.L., White E.A., Tibbs R.E., Haines D.E. (2003). A clinician’s view of spinal cord injury. Anat. Rec. B New Anat..

[27] Pérez R., Martín S., Renán S., Ortiz S.D. (2008). Aspectos epidemiológicos de la lesión medular de la población del Centro Nacional de Rehabilitación. Rev. Mex. Med. Física Rehabil..

[28] De Esclarín Ruz A., Panamericana E.M. (2011). Lesión Medular, Enfoque Multidisciplinario.

[29] Rodríguez Fernández M. (2020). Lesión Medular: Atención Sociosanitaria.

[30] Ahuja C.S., Wilson J.R., Nori S., Kotter M.R.N., Druschel C., Curt A., Fehlings M.G. (2017). Traumatic spinal cord injury. Nat. Rev. Dis. Primers.

[31] Tran A.P., Warren P.M., Silver J. (2018). The biology of regeneration failure and success after spinal cord injury. Physiol. Rev..

[32] Orr M.B., Gensel J.C. (2018). Spinal cord injury scarring and inflammation: Therapies targeting glial and inflammatory responses. Neurotherapeutics.

[33] Schwarz A., Steinstrasser A. (1987). A novel approach to Tc-99m labeled monoclonal antibodies (Abstract). J. Nucl. Med..

[34] Zambrano-Rodríguez P.C., Bolaños-Puchet S., Reyes-Alva H.J., García-Orozco L.E., Romero-Piña M.E., Martinez-Cruz A., Guízar-Sahagún G., Medina L.A. (2019). Micro-CT myelography using contrast-enhanced digital subtraction: Feasibility and initial results in healthy rats. Neuroradiology.

